# *In vitro* Cell Migration, Invasion, and Adhesion Assays: From Cell Imaging to Data Analysis

**DOI:** 10.3389/fcell.2019.00107

**Published:** 2019-06-14

**Authors:** Jordi Pijuan, Carla Barceló, David F. Moreno, Oscar Maiques, Pol Sisó, Rosa M. Marti, Anna Macià, Anaïs Panosa

**Affiliations:** ^1^Flow Cytometry and Confocal Microscopy Unit, IRBLleida, University of Lleida, Lleida, Spain; ^2^IRBLleida, University of Lleida, Lleida, Spain; ^3^Molecular Biology Institute of Barcelona, CSIC, Barcelona, Spain; ^4^Department of Dermatology, Hospital Universitari Arnau de Vilanova, University of Lleida, IRBLleida, Lleida, Spain; ^5^Center of Biomedical Research on Cancer (CIBERONC), Instituto de Salud Carlos III, Madrid, Spain

**Keywords:** cell migration/invasion, wound healing assay, scratch assay, transwell assay, spreading assay, live cell imaging, data analysis, ImageJ/Fiji

## Abstract

Cell migration is a key procedure involved in many biological processes including embryological development, tissue formation, immune defense or inflammation, and cancer progression. How physical, chemical, and molecular aspects can affect cell motility is a challenge to understand migratory cells behavior. *In vitro* assays are excellent approaches to extrapolate to *in vivo* situations and study live cells behavior. Here we present four *in vitro* protocols that describe step-by-step cell migration, invasion and adhesion strategies and their corresponding image data quantification. These current protocols are based on *two-dimensional* wound healing assays (comparing traditional pipette tip-scratch assay vs. culture insert assay), 2D individual cell-tracking experiments by live cell imaging and *three-dimensional* spreading and transwell assays. All together, they cover different phenotypes and hallmarks of cell motility and adhesion, providing orthogonal information that can be used either individually or collectively in many different experimental setups. These optimized protocols will facilitate physiological and cellular characterization of these processes, which may be used for fast screening of specific therapeutic cancer drugs for migratory function, novel strategies in cancer diagnosis, and for assaying new molecules involved in adhesion and invasion metastatic properties of cancer cells.

## Introduction

Cell migration is a crucial process where cells must be able to change and reach their proper position in a given environment to execute their function (te Boekhorst et al., 2016). In multicellular organisms, this phenomenon plays an important role in gastrulation, embryonic morphogenesis, development of the nervous system, tissue homeostasis, and immune cell trafficking. However, moving cells can be deregulated and contribute to many pathological processes such as inflammation and cancer metastasis (Charras and Sahai, 2014; Mayor and Etienne-Manneville, 2016; van Helvert et al., 2018).

In cancer development and progression, invasion, and metastasis occurs when tumor cells disseminate from the primary tumor spreading through the circulatory and lymphatic systems, invade across the basement membranes and endothelial walls and finally colonize distant organs (Friedl and Wolf, 2003; Friedl and Alexander, 2011). Cell migration, invasion, and adhesion are pivotal steps in this process, hence its study, and understanding are crucial in order to fight against the disease. Moreover, cancer cells movement to peripheral organs, and their resultant destruction, constitutes a primary cause of cancer-associated morbidity and mortality (Xu et al., 2018).

Cancer cells use different movement strategies: they can migrate individually or collectively. Individual cell migration is mediated by cytoskeletal activity without cell-cell interactions with neighboring cells, and it has been reported to be important in many *in vivo* physiological processes such as in embryonic developmental stages (Aman and Piotrowski, 2010), immune surveillance (Ridley et al., 2003; Friedl and Weigelin, 2008), and in the early stages of invasion in metastatic process. For example, cancer cells can migrate individually via mesenchymal or amoeboid type of movement. Mesenchymal migration involves integrins and matrix-degrading proteases, while cadherins and cell-cell communication is less relevant in this process (Friedl and Wolf, 2003). In amoeboid migration, typical of lymphocytes and some tumor cells [small-cell lung carcinoma, hematopoietic neoplasias, mammary carcinoma cells (Farina et al., 1998)], the interactions with the substrate are weak. In contrast, collective cell migration occurs as a cohesive cell group that retains cell-cell junctions and coordinates cytoskeletal activity between neighboring cells as well as with the surrounding tissue. It can keep the tissue structure intact and continuous while remodeling it, generating traction, and protrusion forces needed for migration. Collective cell movement allows mobile cells to carry other immobile cell types along and lets migrating cells to influence each other, thereby ensuring appropriate cell distribution and tissue shaping, and also taking collective decisions that would be more robust for the system (Rørth, 2009; te Boekhorst et al., 2016). Among the mechanisms involved there are cell-substrate adhesion and cell-cell adhesion. Cells can strongly adhere to surfaces coated with extracellular matrix (ECM) proteins via focal adhesions complexes and associated stress fibers (Stuelten et al., 2018).

Approaches to study cell migration, invasion, and adhesion are particularly interesting in the fields of physiology and oncology, as they are relevant phenotypes when studying the effect of novel therapeutic drugs and chemoattractants during metastatic progression. Nevertheless, current methods are not efficient enough for *in vitro* high-throughput screening of small molecules and characterization of the molecular metastatic cascade complex. Most of the studies on cell migration have the limitation of being studies based on endpoint assays. New *in vitro* time-lapse microscopy approaches, complex metrics analysis, and downstream interpretation of the motility findings represent a daunting but essential challenge for researchers.

In this report we aim to explain four different *in vitro* protocols for assessing cell migratory behavior and emphasize the importance of an accurate data analysis. In the wound healing collective migration cell protocol, we compare the use of culture insert and the conventional scratch assay using pipette tip, both using time-lapse microscopy approach. All data is processed to determine the migration ability of whole cell masses such as wound area closure, cell front velocity and healing speed. In the individual cell migration approach using time-lapse microscopy, we defined individual cell parameters such as trajectory, accumulated distance, and velocity. The transwell migration/invasion and the spreading assay are used to analyze the ability of single cells to directionally respond to chemoattractants and treatments and to test cell capacity to adhere in the ECM, respectively. All data acquired by these methods is accompanied by a clear explanation on how to proceed in its analysis. Generated images, using build-in ImageJ/Fiji functions and/or some macros could help us to obtain quantitative data from the images in a semi-automated way, which speeds up the quantification step allowing us to analyze larger image sets and obtain more statistically robust results. Combining multiposition time-lapse microscopy with this semi-automated data mining, most of the limitations stated previously could be overcome.

## Materials And Equipment

### Reagents

**CAUTION**. When handling the chemical and biological materials used in this protocol, always wear suitable personal protective equipment, including a lab coat, gloves, safety goggles and, where indicated, a face shield. For any reagent listed in this protocol, appropriate institutional and governmental safety guidelines must be followed. Please refer to the appropriate materials safety data sheets.

#### Human Melanoma Cancer Cell Line and Culture Media

- M3 melanoma cell line (Maiques et al., 2018) or any other cell line of interest and appropriate medium for the cell culture. This protocol has been verified with other melanoma cell lines and non-cancerous cell lines. **CAUTION**. The cell line used in your research should be regularly checked to ensure that they are authentic and they are not infected with *Mycoplasma*.
- Dulbecco's modified Eagle's medium (DMEM), high glucose (4.5 g l^−1^), L-glutamine (2 mM) (Lonza, cat. no. 12-341F; storage at 4°C)
- Penicillin-streptomycin (10,000 U ml^−1^ penicillin and 10,000 μg ml^−1^ streptomycin) (Fisher Scientific, cat. no. W3470H; storage at −20°C)
- Fetal bovine serum (FBS) (Fisher Scientific, cat. no. W3381E; storage at −20°C)
- Fungizone (Fisher Scientific, cat. no. W3474D; storage at −20°C).

#### Other Reagents

- Trypsin-EDTA, 0.25% (wt/vol) (Fisher Scientific, cat. no. W3513C; storage at 4°C)
- Phosphate-buffer saline (PBS), pH 7.4 (see **Reagent Setup**; storage at 4°C)
- HOECHST 33342 (ThermoFisher, cat. no. H3570; storage at 4°C)
- Paraformaldehyde (PFA) (Sigma-Aldrich, cat. no. 158127; storage at −20°C). **CAUTION**. PFA is toxic and corrosive. Avoid any direct contact and wear appropriate personal protective equipment. Use it only inside the chemical hood. Collect and discard waste appropriately.
- Matrigel basement membrane matrix (Cultek, cat.no. 354234; storage at −20°C)
- Fibronectin bovine plasma (Millipore, cat.no. 341631; storage at −20°C)
- Bovine Serum Albumin (BSA) (Sigma-Aldrich, cat. no. A2058; storage at 4°C)
- Mibefradil (Santa Cruz Biotechnology, cat. no. sc-204083A; storage at −20°C)
- Chloroquine (Sigma-Aldrich, cat. no. C6628; storage at −20°C).

### Equipment

#### General Consumables and Equipment

- Sterile 100 mm culture dishes (or other size) (Corning, cat. no. 353003)
- Sterile 24-well plates for cell culture (Corning, cat. no. 353047)
- μ-Dish ^35mm^, high culture insert, tissue culture treated (Ibidi, cat. no. 81176) (examples of other insert suppliers include Cell Biolabs, cat. no. CBA-120)
- μ-Dish ^35mm^, high, tissue culture treated (Ibidi, cat. no. 81156)
- Transwell inserts for 24-well plates (membrane 8.0 μm pores) (Corning, cat. no. 353097)
- Adjustable pipettes: P-2 (Gilson, cat. no. FA10001M), P-20 (Gilson, cat. no. FA10003M), P-200 (Gilson, cat. no. FA10005M), and P-1,000 (Gilson, cat. no. F123602M)
- Pipette tips: 10 μl (Starlab, cat. no. S1121-3810), 20 μl (Starlab, cat. no. S1120-1810), 200 μl (Starlab, cat. no. S1120-8810), and 1,000 μl (Starlab, cat. no. S1120-1830)
- Pipette controller (Corning, cat. no. 357469)
- Tissue culture pipettes: 5 ml (Corning, cat. no. CORN4051), 10 ml (Corning, cat. no. CORN4101)
- Sterile conical (micro) centrifuge tubes: 1.5 ml (Corning, cat. no. 3621), 15 ml (Corning, cat. no. 430052), and 50 ml (Corning, cat. no. 352070)
- Hemocytometer (Sigma-Aldrich, cat. no. Z359629)
- Confocal microscope: inverted microscope (Olympus, model IX-81) with confocal head (Olympus, FluoView 1000); there is no need for confocality, just an automated stage and CO_2_/temperature are needed.
- Microscope cage incubator and CO_2_/temperature controllers (Okolab)
- Cell culture incubator (CO_2_ at 5%, humidified at 37°C) (Thermo Scientific cat. no. 311)
- Laminar flow hood (Telstar, model AV-100)
- Water bath (Thermo Scientific cat. no. 152-4101)
- Forceps (Sigma-Aldrich, cat. no. F4142)
- Cotton swabs (Sigma-Aldrich, cat. no. Z699365).

#### Bioinformatics Tools and Plugins

- Standard PC (with at least 8GB of RAM) running a spreadsheet software (e.g., Microsoft Excel version 2007 or later).
- Fiji or ImageJ: http://fiji.sc/ or https://imagej.nih.gov/ij/h
- Manual tracking: https://imagej.nih.gov/ij/plugins/track/track.html
- Chemotaxis and migration tool or Chemotaxis tool: http://ibidi.com/fileadmin/products/software/chemotaxis_tool/chemotaxis_tool.jar or http://ibidi.com/software/chemotaxis_and_migration_tool/?x504f5=f74e46e10dfbebe758db0683f9de1303
- Cell Counter: http://rsbweb.nih.gov/ij/plugins/cell-counter.html
- New macros provided with this work: Wound_healing.ijm; Wound_healing_Choose_Threshold.ijm; NotSpread&All_.ijm (Supplementary Data 1–3).

### Reagents Setup

#### Fungizone Solution

Prepare a 10 mg ml^−1^ solution. Aliquot and store it at −20°C.

#### Medium for Human Melanoma Cancer Cells Culture

For M3 cells, prepare high glucose (4.5 g l^−1^) containing DMEM supplemented with 100 U ml^−1^ penicillin, 100 μg ml^−1^ streptomycin, 0.1% fungizone and 10% (vol/vol) FBS. Store it at 4°C. **CRITICAL**. Pre-warm the culture medium at 37°C before use.

#### 1X PBS (pH 7.40) Preparation

It contains 137 mM sodium chloride (NaCl) (Sigma-Aldrich, cat. no. 746398), 2.7 mM potassium chloride (KCl) (Sigma-Aldrich, cat. no. P9333), 10 mM disodium hydrogen phosphate (Na_2_HPO_4_) (Sigma-Aldrich, cat. no. 255793) and 1.8 mM potassium dihydrogen phosphate (KH_2_PO_4_) (Sigma-Aldrich, cat. no. P0662). Prepare the buffer in distilled water (dH_2_O), adjust the pH to 7.4 and autoclave it at 121°C, 15psi for 20 min. Store it at 4°C.

#### PFA Fixation Solution, 4% (wt/vol) Preparation

Dissolve 4 g PFA in 100 ml PBS. To dissolve the PFA, heat the solution to ~70°C under constant stirring with a magnetic stirrer in a safety chemical fume hood. Cool the PFA solution, filter it to avoid precipitates in the fixative, aliquot the solution and store it at −20°C.

#### Matrigel Preparation

Thaw aliquots of Matrigel slowly on ice at 4°C overnight. Add 100 μl of Matrigel to a microcentrifuge tube containing 300 μl of cold DMEM (without FBS) (ratio 1:3) and mix thoroughly. **CRITICAL**. Matrigel solution is liquid at 4°C, but it gels at RT. The mixture of Matrigel and DMEM should be prepared freshly every time.

#### Fibronectin Preparation

Prepare a 100 μg ml^−1^ solution (1 ml). Add 100 μl of fibronectin (1 mg ml^−1^) to 900 μl of PBS. Freshly prepare the solution and use it immediately.

#### Heat-Denatured BSA Solution

Dissolve 10 mg of BSA in 1 ml of PBS. Filter the solution through a 0.22 μm filter to remove undissolved protein and incubate it at 90°C for 15 min. The solution can be used after cooling.

## Stepwise Procedeures

**Figure 1:**
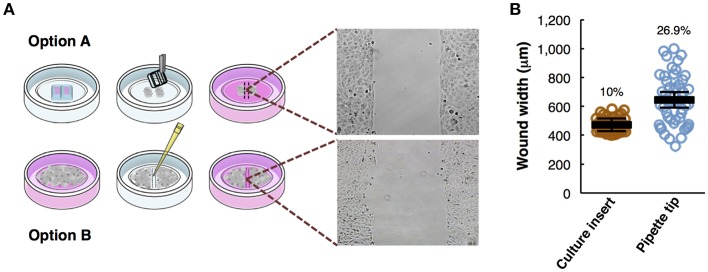
Overview of the wound healing assay preparation protocols. **(A)** Step-by-step scheme showing the differences between wound healing protocol using a culture insert (option **A**) and using pipette tip (option **B**). Phase-contrast microscopy shows gap appearance and both cell fronts just before to start the time-lapse experiment. **(B)** Measurements of wound width (μm) in culture insert (*n* = 50) or pipette tip (*n* = 50). Mean values (thick horizontal lines), confidence limits (α = 0.05, thin horizontal lines), and coefficients of variation (label) are shown.

**Figure 2:**
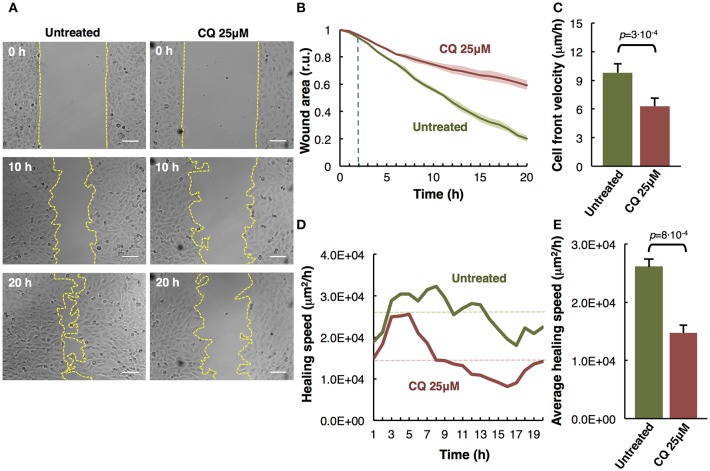
Analysis of M3 melanoma cells migration by *in vitro* wound healing assay. **(A)** Time-lapse microscopy images of wound closure of untreated (left panels) and treated with Chloroquine (CQ 25 μM, right panels) melanoma cells at 0, 10, and 20 h after culture insert removal. The dotted lines define the area lacking cells. Scale bars, 100 μm. **(B)** Quantification of the wounded area invaded during 20 h by untreated (green) and treated with CQ 25 μM (red) melanoma cells presented in relative units (r.u.). Results represent the mean of four measurements of each wounded area, obtained in 3 independent experiments (*n* = 12). Mean values of relative wound closure and corresponding confidence limits (α = 0.05, shaded lines) are plotted. Dotted line marks the time in which significant differences start. **(C)** Analysis of cell front velocity in untreated and CQ 25 μM treated melanoma cells. Mean ± SD from 3 independent experiments (*n* = 12). **(D)** Quantification of healing speed area (μm^2^/h) during 20 h in untreated and treated cells (*n* = 12). Dotted lines mark the average of healing speed area in each treatment. **(E)** Graph showing the average healing speed area (μm^2^/h) quantitative analysis of untreated and treated (CQ 25 μM) melanoma cells; mean ± SD, (*n* = 12), from 3 independent experiments. The corresponding *p-*values obtained by unpaired two-tailed Student's *t*-test are shown in **(C,E)** plots.

**Figure 3:**
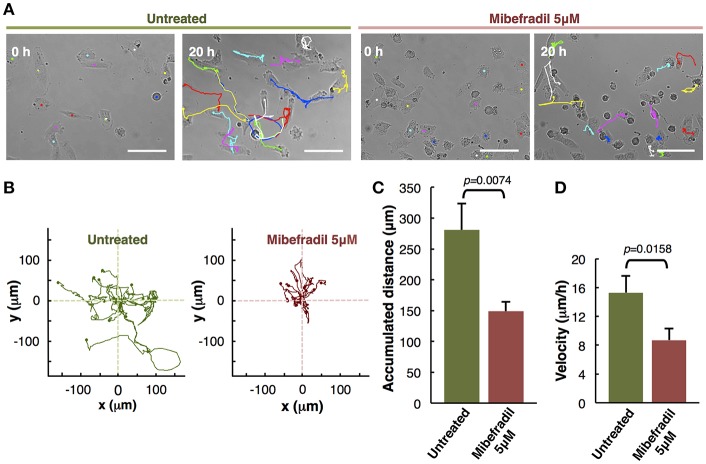
Analysis of individual melanoma cells migration. **(A)** Representative phase-contrast images of untreated and treated with Mibefradil (5 μM) melanoma cells (captured by time-lapse microscopy at 20 min intervals) at initial (0 h) and end (20 h) time. The Manual Tracking plugin of ImageJ/Fiji was used to manually trace 14 representative cell trajectory tracks, marked in colors. Scale bars, 100 μm. **(B)** Trajectory plots showing melanoma cells trajectory during 20 h in untreated (green) and treated (red) cells. All tracks were set to a common origin (intersection of *x* and *y* axes) using Chemotaxis plugin of ImageJ/Fiji. **(C)** Quantitative analysis of average accumulated distance (μm) and **(D)** velocity (μm/h) of untreated and Mibefradil (5 μM) treated melanoma cells. Values are means ± SD (*n* = 5 independent experiments; 300 cells were analyzed for each group). The corresponding *p-values* obtained by unpaired two-tailed Student's *t*-test are shown in **(C,D)** plots.

**Figure 4:**
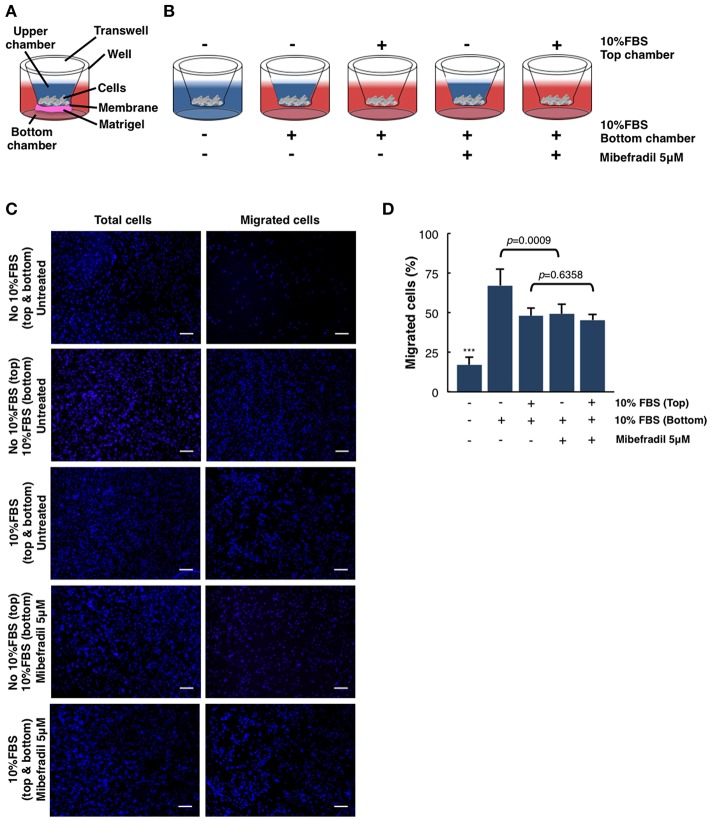
Quantitative and qualitative analysis of melanoma cell migration assessed by *in vitro* transwell assay. **(A)** Schematic illustration of the different parts of transwell system. **(B)** Experimental design scheme of transwell migration assay. Cells were seeded on the upper side of the transwell membrane. In the upper and/or lower compartment, 10% FBS was added as a chemoattractant (red color). When stated, Mibefradil (5 μM) was added in lower compartment. **(C)** Representative fluorescent images of nuclear Hoechst staining (10 μg ml^−1^) were captured at 20 h after treatment indicated total cells (left panel) and migrated cells (right panel). Scale bars, 100 μm. **(D)** Percentage of migrated cells after 20 h, with or without treatment and (–) or (+) chemoattractant (10% FBS). Cells were counted from 10 random microscope fields for each sample in 3 independent experiments. Values are means ± SD. The corresponding *p-values* (*** = *p* < 0.0001) were obtained by one-way ANOVA followed by Tukey-Kramer *post-hoc* test.

**Figure 5:**
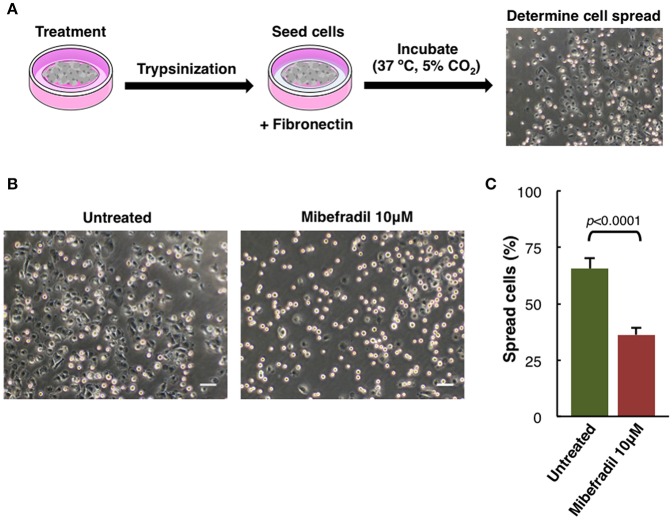
Cell spreading assay in M3 melanoma cells. **(A)** Experimental design scheme of spreading assay. Cells were treated for 24 h, trypsinized and seeded onto fibronectin (10 μg ml^−1^) coated plate. After 1 h cells were fixed with 2% PFA and **(B)** phase-contrast images were captured. Scale bars, 50 μm. **(C)** Plot showing the percentage of spreading cells. Round bright cells were considered unspread. Values are percentage of spread cells ± SD (*n* = 3 independent experiments; at least 600 cells for each experiment were counted). The corresponding *p-*value obtained by unpaired two-tailed Student's *t*-test is shown.

**1. Cell Culture [TIMING ~ 4 Days]**
**1.1**) Seed cells into 100 mm culture dishes at 25–30% confluence, and grow them for 3 days (at 37°C and 5% CO_2_) in appropriate culture medium (e.g., M3 cells, use DMEM with 10% (vol/vol) FBS [see **Reagent Setup**)].
**1.2**) Remove medium from culture dish by gentle aspiration, wash cells twice with sterile 1X PBS, add trypsin-EDTA enough to completely cover the cells and place them at 37°C for 2 min. After incubation, add an equal volume of complete medium to stop the trypsin-EDTA reaction and collect all the liquid in a sterile tube. Centrifuge the cell suspension for 5 min at 300 g at RT, then remove the trypsin-EDTA solution by aspiration and mix the cells with fresh medium containing serum. Determine cell concentration using a hemocytometer.
**? Troubleshooting**
**1.3**) (Optional step, depending on the cell type used) Coating dishes with extracellular matrix (ECM) substrates (e.g., gelatin, collagen, or fibronectin) in order to adhere the cells and incubate it at 4°C overnight.
**? Troubleshooting**
**2. Wound Healing Assay Using Silicone Insert vs. Scratch Assay Using Pipette Tip**
**2.1**) Seed the cell suspension into each well with the silicone culture insert on 35 mm culture insert μ-dish to perform wound healing assay (option A), or alternatively seed cells onto all surface of 35 mm culture μ-dish to perform scratch assay (option B) (see scheme on Figure 1A). Incubate the dishes for 6 h or overnight at 37°C and 5% CO_2_, allowing cells to adhere and spread on the substrate. The number of cells to create a confluent monolayer depends on your cell type and the size of dishes. You have to adjust the characteristics depending on these parameters [e.g., using M3 cells, seed 3·10^4^ cells/well when using the silicone culture insert (area for each well = 0.22 cm^2^) and seed 4.5·10^5^ cells/dish (area for dish = 3.5 cm^2^)].
**CRITICAL STEP**. To ensure the reproducibility on the results, it is very important to create a confluent cell monolayer (90–95% confluence) and maintain a constant seeding number specific to each cell line.
**2.2**) Follow option **A** to perform the wound healing assay with culture insert, and option **B** to perform scratch assay with pipette tip (Figure 1A).
(**A) *In vitro* wound healing assay (with insert) and data analysis [TIMING ~ 2 days]**
(**i**) Check the cells under the microscope to ensure that a confluent cell monolayer (95–100%) is created.
**? Troubleshooting**
(**ii**) Remove the culture insert with sterile forceps.
**CRITICAL STEP**. Use sterile forceps to grab the insert slowly and gently from the dish. Avoid twisting the insert.
**? Troubleshooting**
(**iii**) Remove debris and non-attached cells by washing the cell layer twice with 1 ml of sterile 1X PBS and then replace it with 2 ml of appropriate medium for the assay, if it is necessary add a treatment to the medium (e.g., metastasis inhibitor Wang et al., 2015, antibiotics Zhu et al., 2016, depending on the aim of the experiment).
**CRITICAL STEP**. Pre-warm the 1X PBS and the medium at 37°C. Avoid detaching cells in the wash.
(**iv**) Place the dish under a phase-contrast scanning confocal microscope with a CO_2_ microscope cage incubator, image acquisition is performed at 37°C and 5% CO_2_. Define exact positions and focal planes, avoiding overlapping fields. Start image acquisition by taking images several times throughout 20–24 h (e.g., time-lapse measurements for cultured M3 melanoma cells were performed for 20 h with a time interval of 1 h and 10X objective). Select 3 or 4 fields from each wound and at least three wounds per sample.
**CRITICAL STEP**. Prior to the time-lapse microscopy setup, switch on the CO_2_ and temperature to equilibrate the system at least 30 min before starting acquisition. Ensure that the parameters are set at 5% CO_2_ and 37°C.
**OPTIONAL STRATEGY**. Fluorescent-tagged cells can be used in this assay, although the segmentation steps prior to wound area quantification should be modified, as right now they are optimized for transmission light images. You must also be careful with light intensity during time-lapse acquisition, as it may cause photo-toxicity.
**? Troubleshooting**
(**v**) After acquiring the time-lapse images, review the frames in a sequence format (^*^.tif, ^*^.oif, ^*^.zvi or equivalent).
**PAUSE POINT**. Data processing can be conducted as per convenience of user.
(**vi**) Quantitative data analysis can be performed with Open-source software (ImageJ/Fiji). In ImageJ/Fiji, time-lapse sequences can be opened by clicking *File* → *Open*.
(**vii**) Calibrate the image sequence by using *Analyze* → *Set scale* and introduce the correct scale (pixels/μm) depending on the objective and microscope used (Supplementary Figure 1A), if this information is not stored automatically by your microscope's software in the data file.
**CRITICAL STEP**. It is important to set the correct scale for the cell migration analysis.
**? Troubleshooting**
(**viii**) Select the measurement parameters *Analyze* → *Set measurements* → *Area* (Supplementary Figure 1A).
(**ix**) Click on duplicate the image sequence *Image* → *Duplicate*.
(**x**) Detect the change intensity on the edge on the duplicate sequence *Process* → *Find edges* (Supplementary Figure 1B).
(**xi**) Apply a blurred edge filter *Process* → *Filters* → *Gaussian Blur* (e.g., Sigma (Radius) = 5) (Supplementary Figure 1C).
(**xii**) Adjust the intensity threshold to detect the wound area *Image* → *Adjust* → *Threshold* (Supplementary Figure 1D).
**CRITICAL STEP**. Correctly define the threshold parameter to determine exactly the empty wound area. By default, the macro is running an AutoThreshold using the Minimum method (Supplementary Data 1), which acceptably operates in most of time-lapse data.
(**xiii**) Create a selection around the threshold area *Edit* → *Selection* → *Create selection*.
(**xiv**) The area selection is first shrunk and then enlarged (e.g., 10 pixels) in order to eliminate small thresholded areas not related with the wound, and then is returned back to the original size in the wound *Edit* → *Selection* → *Enlarge (*e.g., *first plug* −*20, then 20)*.
(**xv**) Add all time-points into ROI manager *Analyze* → *Tool* → *ROI manager*.
*Steps xiii – xv must be repeated for every time-point if done manually (without using the macro)*.
(**xvi**) Obtain the area value in the different time-points *Analyze* → *Measure* (Supplementary Figure 1E).
(**xvii**) We have written the steps between (ix) and (xvi) in a macro for ImageJ/Fiji to automatically analyze the wound healing area in each image sequence. The code “Wound_healing.ijm” is provided in Supplementary Data 1. Execute this code at ImageJ/Fiji and your time-lapse images will be automatically quantified.
**? Troubleshooting**
(**xviii**) Additionally, Click on “*Straight*” button (toolbar) and measure the length (μm) between one side of the scratch and the other in the images acquired at initial (0 h) and end time to determine the cell front velocity. Scratch length measurement should be done in three regions of each image between the closest points on both sides of the wound.
(**xix**) Import the data to spreadsheet software and determine the relative wound area closure, velocity, and other parameters (see **ANTICIPATED RESULTS** and Figure 2). Statistical significance of the differences between samples was estimated by unpaired two-tailed Student's *t*-test, which can be performed straight in the spreadsheet. If your experimental setup includes more than two conditions to compare, you may use more specific software like SPSS, where you could perform more complex analysis by one-way ANOVA followed by Tukey-Kramer *post-hoc* test.
(**B) *In vitro* scratch assay (with pipette tip) and data analysis [TIMING ~ 2 days]**
(**i**) Continuing from step 2.2 (option **B**), make a wound by scratching the cell monolayer in a straight line with a sterile P-200 pipette tip.
**CRITICAL STEP**. Scratching should be done gently with a P-200 pipette tip in a straight line in the center of the dish to only detach central cells. Try to move the tip in a continuous way and always with a similar size in each well to avoid variation between conditions.
**? Troubleshooting**
(**ii**) Wash twice with 1 ml of sterile 1X PBS to remove the floating cells and debris and add 2 ml of appropriate medium for the assay (if it is necessary add a treatment in the medium).
**CRITICAL STEP**. Pre-warm the 1X PBS and the medium at 37°C. Avoid detaching cells in the wash.
(**iii**) Repeat steps 2.2(A) (iv-v) for phase-contrast scanning setup and acquisition parameters.
(**iv**) Quantitative data analysis. Repeat steps 2.2(A) (vi)–(xvii). In ImageJ/Fiji, open the individual images and perform the same procedure.
**? Troubleshooting**
(**v**) Additionally, Click on “*Straight*” button (toolbar) and measure the length (μm) between one side of the scratch and the other in the images acquired at initial (0 h) and end time to determine the cell front velocity. Length scratch measurement should be done in three regions of each image between the closest points on both sides of the wound.
(**vi**) Import data to spreadsheet software and proceed as explained in the step 2.2(A) (xix).
**3. Individual Cell-Tracking Assay and Data Analysis [TIMING ~ 2 Days]**
**3.1**) Continuing from step 1.2 (or optionally step 1.3), seed 1.2·10^4^ cells in 500 μl of proper complete medium onto each well on 24-well plates and grow them overnight (at 37°C and 5% CO_2_) (e.g., for M3 melanoma cells, use DMEM with 10% (vol/vol) FBS (see **Reagent Setup**) and perform at least three biological replicates for each different experimental condition).
**CRITICAL STEP**. To analyze individual cell-tracking, cells are seeded at low density. The number of seeded cells depends on the characteristics of the cell type and the size of dishes.
**? Troubleshooting**
**3.2**) Carefully remove the debris and dead cells by washing the wells once with 500 μl of sterile 1X PBS and then replace it with 500 μl of proper medium for the assay. Add a specific treatment in the medium, if necessary.
**3.3**) Immediately place the 24-well plate under the phase-contrast or fluorescence microscope with a CO_2_ microscope cage incubator (at 37°C and 5% CO_2_). Perform time-lapse imaging (e.g., taking an image every 20 min for 20 h using 20X objective). You should acquire about 10–15 fields for each of the three biological replicates in order to obtain data from at least 300 cells.
**CRITICAL STEP**. Define the image acquisition setup depending on the previous knowledge about your cell line (e.g., higher temporal resolution if your cell line is known to have high motility). Avoid overlapping fields.
**CRITICAL STEP**. Prior to time-lapse microscopy setup, switch on the CO_2_ and temperature to equilibrate the system at least 30 min before starting acquisition. Ensure that the parameters are set at 5% CO_2_ and 37°C.
**OPTIONAL STRATEGY**. Fluorescent-tagged cells can be used in this assay. You must also be careful with light intensity during time-lapse acquisition, as it may cause photo-toxicity.
**? Troubleshooting**
**3.4**) Quantitative data analysis. Repeat steps 2.2(A) (v)-(vii).
**3.5**) Open *Plugins* → *Tracking* → *Manual Tracking*.
**3.6**) Set the parameters “*Time interval*,” “*x/y calibration*,” and “*z calibration*” in the *Manual Tracking* (Supplementary Figure 2A).
**? Troubleshooting**
**3.7**) Start individual cell-tracking analysis, click on “*Add track*” and then select one cell in the first time-point and follow it through all time-points, finally click on “*End track*.” Repeat it for all the cells in the frame through all the time-points. A new window with the results (*track, slice, distance* and *velocity*) appears (Supplementary Figure 2B).
**3.8**) Save the values in (.txt) format and click on “*Overlay dots and line*” to save the sequence with the track.
**3.9**) Download Chemotaxis plugin and open it in ImageJ/Fiji *Plugins* → *Chemotaxis tool*. Alternatively, the “*Chemotaxis and migration tool*” software can be downloaded and used with the same function (see **EQUIPMENT**).
**3.10**) Set parameters. Click on “*Settings*” and apply the correct settings for “*Time interval*” and “*x/y calibration*” (Supplementary Figure 2C).
**? Troubleshooting**
**3.11**) Import dataset. Click on “*Import data*” and select the (.txt) file with the obtained values in Manual Tracking plugin. Select the file, set the number of slices to analyze and click on “*Add dataset*.” Select up to four datasets and work simultaneously on this plugin (Supplementary Figure 2D). Optionally add some restrictions in the parameters. Click on “*Open restrictions*” and “set *split dataset*,” “*threshold distance*,” and “*threshold velocity*.”
**3.12**) Click on “*Apply settings*.”
**3.13**) Click on “*Plot feature*” select no marks (or appropriate marks), adjust the axis scaling and click on “*Plot graph*” to obtain the trajectory plot. Optionally, an animated trajectory plot can be obtained.
**3.14) (**Optional step, depending on the analysis). Click on “*Diagram feature*” and select “*Plot histogram*,” “*Plot rose diagram*,” or “*Circular plot*” to obtain several plots that show an orientation/distribution of all cell migration directions.
**3.15**) Click on “*Statistics feature*” and select “*velocity*,” “*distance”* (euclidean and accumulated), “*FMI”* (Forward Migration Index) and “*directionality*.” Save or export these data to spreadsheet software.
**3.16**) In the spreadsheet software, determine the mean and standard deviation (SD) of velocity (μm/h), accumulated distance (μm), euclidean distance (μm) and others parameters (see **ANTICIPATED RESULTS** and Figure 3). Statistical significance of the differences between samples was estimated by unpaired two-tailed Student's *t*-test, which can be performed straight in the spreadsheet. If your experimental setup includes more than two conditions to compare, you may use more specific software like SPSS, where you could perform more complex analysis by one-way ANOVA followed by Tukey-Kramer *post-hoc* test.
**4. Transwell Cell Migration/Invasion Assay and Data Analysis [TIMING ~ 2 Days]**
**4.1**) This assay can be used to analyze cell migration and/or invasion. To analyze cell invasion, the transwell insert membrane is coated with Matrigel while in cell migration assays it is not. This procedure is identic for both possibilities; the only difference is the presence or not of Matrigel. (If you want to do a migration assay omit the Matrigel steps).
**4.2**) Matrigel step: Thaw the Matrigel solution at 4 °C overnight. Coat the down side surface of the transwell membrane with 25 μl of Matrigel solution (see the preparation in **Reagent Setup**, Figure 4A).
**CRITICAL STEP**. Matrigel solution is liquid at 4°C, but it gels very quickly at RT. Remember to work in cold conditions and previously chill at −20°C all the material (pipettes, tips, and forceps).
**4.3**) Incubate the transwell with Matrigel at 37°C for 30 min for gelling.
**? Troubleshooting**
**4.4) (**Optional step) Wash off the Matrigel of the down side of the membrane twice with pre-warmed serum-free culture medium (e.g., for M3 melanoma cells culture use DMEM medium without FBS).
**CRITICAL STEP**. Gentle washes. Avoid detaching Matrigel in the washes.
**4.5**) Add 500 μl of culture medium (with or without chemoattractant) into each well on 24-well plate (e.g., for melanoma cells transwell assay, 10% FBS was used as a chemoattractant. See scheme Figure 4B).
**4.6**) (Optional step) Add a treatment in the culture medium if necessary.
**4.7**) Use sterile forceps to transfer the transwell insert into each well of the 24-well plate already filled with culture medium (with or without chemoattractant).
**4.8**) Continue from step 1.2, add 400 μl of cell suspension (at final confluence 50–60%) onto the transwell upper chamber (see scheme Figure 4A).
**CRITICAL STEP**. Depending on your cell type size, the required pore size of the transwell membrane insert may change (e.g., in this protocol 8 μm pore diameter membranes are used).
**4.9**) Incubate at 37°C and 5% CO_2_ for 20–24 h.
**4.10**) Fix cells by adding paraformaldehyde (PFA) at final concentration 4% in culture medium into both sides of each transwell insert for 15 min at RT. **CAUTION**. Perform cell fixation in a safety chemical fume hood, and wear appropriate personal protective equipment.
**4.11**) Remove culture medium with 4% PFA by gentle aspiration.
**4.12**) Wash transwell insert twice at both sides of the membrane with sterile 1X PBS to remove debris, non-attached cells, and fixation solution excess.
**CRITICAL STEP**. Be gentle with all washes to avoid detaching cells.
**PAUSE POINT**. Samples can be stored in 1X PBS at 4°C for up to 1 month.
**4.13**) Stain cells, by incubating the transwell insert (with or without Matrigel) with Hoechst (final concentration 10 μg ml^−1^ in 400 μl of 1X PBS) for 15 min at RT protected from light. (Crystal violet or Hematoxylin can also be used).
**CRITICAL STEP**. From this step until step 4.18 work in dark conditions.
**4.14**) Wash the transwell insert twice with sterile 1X PBS to remove Hoechst solution excess.
**4.15**) Maintain inserts in the 24-well plate with sterile 1X PBS to avoid drying the membrane.
**4.16**) Capture several images in order to cover up all the surface of the transwell insert membrane, either with Matrigel coating or not, using 10X or 4X objectives on a fluorescence microscope. To avoid overlapping fields, an automatic tiling function when acquiring images may be used (if it is available in your equipment). The number of cells counted using these images will be the total number of cells in invasion assay (if Matrigel was used) or the total number of cells in migration assay (if Matrigel was not used).
**4.17**) Immediately remove non-invaded/migrated cells from the upper surface of the transwell membrane by gently scrapping with a cotton swab. If Crystal violet or Hematoxilin is used the membrane turns blue. Scrap the membrane surface until the last swab used remains white (or clean).
**CRITICAL STEP**. Be careful with scrapping. Scrapping must be gentle with little pressure to remove all the cells on the upper surface of the membrane but not affecting the migrated/invaded cells of the bottom. If it is necessary, repeat the scrapping with a second cotton swab.
**? Troubleshooting**
**4.18**) Repeat step 4.16, but in this case the acquired images represent the invaded or migrated cells (with or without Matrigel, respectively).
**4.19**) Quantitative data analysis can be performed with Open-source software (ImageJ/Fiji). In ImageJ/Fiji, acquired images can be opened by clicking *File* → *Open*.
**4.20**) Select *Process* → *Find Maxima* to measure automatically the total number of cells in each image.
**? Troubleshooting**
**4.21**) Adjust the parameter “*Noise tolerance*” to avoid background noise, detect, and count all cells (nuclei stained by Hoechst) (Supplementary Figure 3A).
**? Troubleshooting**
**4.22) (**Alternatively to step 4.20–4.21), a manual counting can be obtained by opening *Plugins* → *Analyze* → *Cell counter*.
**4.23**) Select “*Initialize”* and “*Type I”* and click on the cells (nuclei stained by Hoechst) manually to obtain the total number of cells (Supplementary Figure 3B).
**4.24**) Import data to the spreadsheet software and determine the percentage of migrated and/or invaded cells (see **ANTICIPATED RESULTS** and Figure 4). Statistical significance of the differences between samples was estimated by unpaired two-tailed Student's *t*-test, which can be performed straight in the spreadsheet. If your experimental setup includes more than two conditions to compare, you may use more specific software like SPSS, where you could perform more complex analysis by one-way ANOVA followed by Tukey-Kramer *post-hoc* test.
**5. Spreading Assay and Data Analysis [TIMING ~ 2 Days]**
**5.1**) Continue from step 1.2, seed 5·10^4^ cells in 500 μl of proper medium for the cell line used onto each well on 24-well plates and grow them overnight or at least 6 h (at 37°C and 5% CO_2_), allowing cells to adhere (e.g., for M3 cells, use DMEM with 10% (vol/vol) FBS (see **Reagent Setup**). Perform at least three biological replicates for each different experimental condition).
**CRITICAL STEP**. Cells are seeded at 60–70% confluence. The number of seeded cells depends on the cell type and the size of dishes.
**5.2**) Remove medium from each well by gentle aspiration, wash the cells once with sterile 1X PBS and add 500 μl of medium (with or without treatment).
**5.3**) Incubate at 37°C and 5% CO_2_ for 24 h.
**CRITICAL STEP**. The incubation period depends on the treatment and the experiment.
**5.4**) Coat the wells of a new 24-well plate with 250 μl of fibronectin (10 μg ml^−1^) and incubate for 1 h at 37°C or overnight at 4°C (see the preparation in **Reagent Setup**). Other extracellular matrix (ECM) substrates can be used e.g., gelatin or collagen, depending on your cell line.
**? Troubleshooting**
**5.5**) Remove the unbound fibronectin by gentle aspiration and wash twice with sterile 1X PBS.
**5.6**) (Optional step to block chemoattractant proteins present in the extracellular matrix coating). Add 200 μl of heat-denatured BSA solution (10 mg ml^−1^) into each well, incubate for 30 min at RT or overnight at 4°C and wash twice with sterile 1X PBS (see the preparation in **Reagent Setup**).
**5.7**) After step 5.3, remove medium (with or without treatment) from each well by gentle aspiration, wash cells once with sterile 1X PBS and add trypsin-EDTA to completely cover the cells. Place them at 37°C for 2 min. Add equal volume of complete medium to stop the trypsin-EDTA reaction. Centrifuge the cell suspension for 5 min at 300 g at RT, and then remove the trypsin-EDTA and complete media solution by aspiration. Mix cells with 500 μl of fresh medium containing serum.
**5.8**) Seed 2·10^4^ cells in 500 μl of proper medium (according to cell line used) onto the fibronectin coated 24-well plate (step 5.5).
**? Troubleshooting**
**5.9**) Incubate at 37°C and 5% CO_2_ for 1 h.
**CRITICAL STEP**. The incubation period depends on the cell type and the treatment used. This cell type-specific binding time must be determined previously by the researchers.
**? Troubleshooting**
**5.10**) Fix cells. Add PFA in the medium (final concentration 2%) of each well for 15 min at RT.
**CAUTION**. Perform the cell fixation in a safety chemical fume hood, and wear appropriate personal protective equipment.
**5.11**) Remove culture medium containing 2% PFA by gentle aspiration.
**5.12**) Wash the wells twice with sterile 1X PBS to remove debris, non-attached cells, and fixation solution excess.
**CRITICAL STEP**. Be gentle with all washes to avoid detaching the cells.
**PAUSE POINT**. Samples can be stored in 1X PBS at 4°C for up to 1 month.
**5.13**) Acquire several images of fixed cells in a phase-contrast microscope using 10X objective and avoid overlapping fields.
**5.14**) Quantitative data analysis can be performed with Open-source software (ImageJ/Fiji). In ImageJ/Fiji, acquired images can be opened by clicking *File* → *Open*.
**CRITICAL STEP**. In order to be analyzed, images must be 8-bit. If they are in a different bit depth, modify them by clicking *Image* → *Type* → *8-bit*.
**5.15**) Image background was substracted using a rolling ball of 10px radius by clicking *Process* → *Substract background*. Afterwards, the image was duplicated by clicking *Image* → *Duplicate*. One image will be used to quantify the unspread cells, while in the other one we will quantify all the cells.
**5.16**) To quantify unspread cells (Supplementary Figure 4A), the image was thresholded by the Yen method, just leaving the brightest sections of the image (corresponding to unspread cells) by clicking *Image* → *Adjust* → *Threshold* and selecting the Yen method. This creates a binary image. Afterwards, the image was inverted by clicking in *Edit* → *Invert*.
**5.17**) Some binary operations, like “Erode,” “Dilate,” and “Fill Holes” were performed in order to polish the thresholding; you can find them in *Process* → *Binary* → … Later, the watershed algorithm was used to segment cells that were touching each other, by clicking *Process* → *Binary* → *Watershed*.
**5.18**) Particle number was counted automatically by clicking *Analyze* → *Analyze particles*, setting a minimal size of 100px^2^ (as smaller particles were just little remains of other features coming from the thresholding, not real unspread cells).
**5.19**) To quantify all cells (Supplementary Figure 4B), the duplicated image created in step 5.15 was used. First, to better detect the spread cells, we highlighted all the cell edges by clicking *Process* → *Find Edges*. Afterwards, the image was inverted by clicking in *Edit* → *Invert*.
**5.20**) The image was thresholded using the Trinagle method, less restrictive than the Yen method but still selecting where the cells are, by clicking *Image* → *Adjust* → *Threshold* and selecting the Triangle method, creating a binary image.
**5.21**) Some binary operations, like “Erode,” “Dilate,” and “Fill Holes” were performed in order to polish the thresholding, you can find them in *Process* → *Binary* → … Later, the watershed algorithm was used to segment cells that were touching each other, by clicking *Process* → *Binary* → *Watershed*.
**5.22**) Particle number was counted automatically by clicking *Analyze* → *Analyze particles*, setting a minimal size of 100px^2^ (as smaller particles were just little remains of other features coming from the thresholding, not real cells).
**5.23**) We have written the steps between 5.15 and 5.22 in a macro for ImageJ/Fiji to automatically analyze and determine the number of unspread and total cells in the image. The code “Unspread&All_.ijm” is provided in Supplementary Data 3. Execute this code at ImageJ/Fiji and your images will be automatically quantified.
**CRITICAL STEP**. The macro may have troubles in identifying the total number of cells if the cells are seeded too confluent; it is better to seed at lower density and take images of a few more fields in order to ensure a reliable quantification. Otherwise, the analysis may be done manually (*Plugins* → *Analyze* → *Cell counter*), but this will slow down the quantification a lot, and still it is hard to identify the boundary between cells if the culture is too confluent.
**CRITICAL STEP**. Round and refringent cells were considered unspread, while dark cells with cytoplasm surrounding the entire circumference of the nucleus were considered spread cells. The sum of spread and unspread cells is the total number of cells. We can infer the number of spread cells from the difference between total number of cells and unspread cells.
**5.24**) Import data to the spreadsheet software and determine the percentage of spread cells to total cells (see **ANTICIPATED RESULTS** and Figure 5). Statistical significance of the differences between samples was estimated by unpaired two-tailed Student's *t*-test, which can be performed straight in the spreadsheet. If your experimental setup includes more than two conditions to compare, you may use more specific software like SPSS, where you could perform more complex analysis by one-way ANOVA followed by Tukey-Kramer *post-hoc* test.

## Timing

### 1. Cell Culture

Step 1.1: 2 d

Step 1.2: 15–20 min

Step 1.3 (optional): overnight

### 2(A). Wound Healing Assay and Data Analysis

Step 2.1: overnight

Steps 2.2(A) (i–iii): 20–30 min

Step 2.2(A) (iv): 20–24 h (as needed)

Steps 2.2(A) (v-xix): 3–4 h (as needed)

### 2(B). Scratch Assay and Data Analysis

Steps 2.2(B) (i–ii): 15–30 min

Step 2.2(B) (iii): 20–24 h (as needed)

Step 2.2(B) (iv-vi): 3–4 h (as needed)

### 3. Individual Cell-Tracking Assay and Data Analysis

Steps 3.1–3.2: 20 min

Step 3.3: 20–24 h (as needed)

Steps 3.4–3.8: 2–5 h (as needed)

Steps 3.9–3.16: 3–5 h (as needed)

### 4. Transwell Cell Migration/Invasion Assay and Data Analysis

Step 4.2: overnight

Step 4.3: 30 min

Steps 4.4–4.6: 15 min

Steps 4.7–4.8: 15 min

Step 4.9: 20–24 h (as needed)

Step 4.10: 15 min

Steps 4.11–4.12: 10 min

Step 4.13: 15 min

Steps 4.14–4.15: 10 min

Step 4.16: 10 min

Step 4.17: 10 min

Step 4.18: 10 min

Steps 4.19–4.24: 3–5 h (as needed)

### 5. Spreading Assay and Data Analysis

Step 5.1: overnight (as needed)

Step 5.2: 10 min

Step 5.3: 24 h (as needed)

Step 5.4: overnight (as needed)

Step 5.5: 5 min

Step 5.6 (optional): 30 min

Step 5.7: 15 min

Step 5.8: 20 min

Step 5.9: 1 h

Steps 5.10–5.12: 30 min

Steps 5.13–5.24: 3–5 h (as needed)

## Anticipated Results

To analyze the migratory modalities and discern phenotypic cell behavior during the process, we conducted four different *in vitro* assays and the corresponding image analysis quantification. Some steps in these protocols can be improved based on instructions provided in Table 1.

**Table 1:** Troubleshooting table (?).

**Step**	**Problem**	**Possible reason**	**Solution**
1.2	Low cell viability	Excessive trypsinization	Decrease the trypsinization time or trypsin concentration
1.3	Low cell adhesion	The cell line needs ECM coated dishes	Choose a proper ECM for the cell line
2.2(A) (i) and 2.2(B) (i)	Low cell density and no cell monolayer	Insufficient cell seeding density in step 2.1	Seed more cells in step 2.1. Adjust it depending on the cell line
2.2(A) (ii)	Wound edges are not straight	Culture insert virulently removed	Grab the culture insert in the corner and remove it slowly and gently
2.2(A) (iv) and 3.3	Cells grow poorly inside the field of view in the microscope	Photo-toxicity	Increase the frame intervals or reduce the amount of excitation light
2.2(A) (vii), 2.2(B) (iv), 3.6 and 3.10	Unknown pixel/μm ratio	Capture the images without indicating pixel/μm parameters in this augment	Use a calibration slide
			Look for information about the microscope and augment used
2.2(A) (xvii)	The wound area is not selected properly	The thresholding parameter is not working correctly in your data	We have developed a second version of this macro which executes all available threshold methods on ImageJ/Fiji, allowing the user to select the one which has a better performance, at the cost of slowing down data results (see Supplementary Data 2). If the user wishes to permanently change the thresholding method in the simpler version of the macro (Supplementary Data 1), it can be done by substituting it in the line 20 of the macro: setAutoThreshold (“yourThreshold”).
	The macro only thresholds the first time-point when using formats different from .tif	You are using the ‘virtual stack' in the Bio-formats Import Options	Untick this option when loading the image, or convert it to .tif before quantification
2.2(B) (i)	The scratch area is irregular, non-well defined	Scraping is done slowly and bent	Scrape the cells in a straight line, with moderate speed, always applying the same pressure Scrape the cells with other tip size, P-2 o P-1,000 depending of the need.
	Cells do not grow onto the scratch area	Scraping is done too hard and the ECM is removed	Scrape more slowly and gently. Detach only the cells
3.1	High cell density	Excess of cells are seeded in step 3.1	Seed less cell density in step 3.1. To analyze individual cell-tracking a low density is necessary
4.3	Matrigel is not gelled	37°C or RT is recommended to gel the Matrigel. At low temperature it is liquid	Incubate the transwell with Matrigel at 37°C at least 30 min
4.17	Not all non-invaded cells have been removed	The scrape done with a cotton swab does not remove all non-migrated cells (cells on the upper surface)	Repeat the scrape with a cotton swab as many times as necessary until non-invaded cells are removed
4.20 and 4.21	High levels of noise in the images	Saturate image	Adjust the “*Noise tolerance*” to count the exact number of cells and avoid a false estimation
5.4	Fibronectin (or another ECM) shows an irregular pattern on culture dishes	The coated dishes were placed in an irregular surface	Coat the dishes on a smooth surface and avoid vibrating sources
5.8	Clump of cells in the center of the well	The plate was not swirled when cells were seeded	Swirl the plate after seeding the cells
5.9	All cells are spreading in the fibronectin surface	The incubation period in step 5.9 is too long	Reduce incubation time. Check the cells regularly under the phase-contrast microscope
	Any cells are spreading in the fibronectin surface	The incubation period in step 5.9 is too short	Increase incubation time. Check the cells regularly under the phase-contrast microscope

### Comparison of Wound Healing Assay Using a Culture Insert *vs*. a Pipette Tip Scratch

Wound healing assays, either using a culture insert or a traditional pipette tip (scratch assay), are two variants of a technique used to analyze collective cell migration. Both methods described are schematically depicted (Figure 1A). Option A shows the use of culture insert to create two cell fronts, and option B shows the pipette tip scratched cells. To compare the accuracy and robustness of both techniques, we measured the wound width (*n* = 50) produced by both methods. In the culture insert assay, the variation coefficient is significantly lower (10%) than with the pipette tip scratch (26.9%), almost increasing 3-fold the variability on the initial conditions of the experiment (Figure 1B).

The technique is effective analyzing cell migration, but there are some advantages and disadvantages specific for each case: while the culture insert allows the user to increase reproducibility between replicates, yielding more reliable and accurate results, the pipette tip scratch is a much cheaper and faster method.

### The Effect of Chloroquine in Collective Cell Migration

Cell migration is a key process during melanoma metastasis. We assessed the effect of Chloroquine (CQ) treatment, that blocks autophagy preventing autophagosomes fusion with lysosomes (Boya et al., 2005; Maiques et al., 2018) in M3 melanoma collective cell migration using an *in vitro* wound healing assay employing culture inserts.

M3 cells were treated at initial time and compared to untreated cells during 20 h by time-lapse imaging. The images of three representative collective cell migration time-points are shown: start (0 h), middle (10 h), and end (20 h) (Figure 2A). Different types of quantitative analysis were performed to determine the effect of the CQ (25 μM) treatment over the melanoma cells collective migration. Wound area relative closure was determined between treated and untreated cells during 20 h using time-lapse live imaging and the images were quantified using the macro “Wound_healing.ijm” in ImageJ/Fiji (Supplementary Figure 1 and provided in Supplementary Data 1) to analyze the invaded area in each time-point. We observed a significant slower collective cell migration in M3 cells treated with CQ (red) compared to untreated cells (green) (Figure 2B). Alternatively, the cell front velocity (μm/h) was measured, as complementary information, between initial and end time-point. To determine this parameter in untreated and CQ treated melanoma cells, the distance between cell front and the half of the wound width was measured at 0 h and 20 h time-points. The equation for cell front velocity (*i*) was applied. We observed a significant difference (*p* = 3·10^−4^) between untreated (9.80 ± 0.92 μm/h) and treated cells (6.28 ± 0.85 μm/h) (Figure 2C).

$$\begin{array}{l} \left(i\right)\;v=\frac{\text{Distance}\;\text{in}\;\text{initial}\;\text{time}\;\left(\mu m\right)\text{ Distance}\;\text{in}\;\text{final}\;\text{time}\;(\mu m)}{\;\text{Total}\;\text{time}\;(h)} \end{array}$$

Other parameters to measure were the healing speed and the average healing speed (μm^2^/h) during 20 h. To measure the healing speed inside the wound area in each time-point, the general equation for a straight line (*y* = *mx*+*b*) was used, where *m* is the slope of the line. The slope between each time-point was measured and the average in each hour of all biological replicates and experiments was plotted for untreated and treated cells. We perceive a decrease of healing speed in cells treated with CQ compared with untreated cells in each time-point (Figure 2D). The healing speed average in three independent experiments shows a significant decrease (*p* = 8·10^−4^) in CQ treated M3 cells (1.47·10^4^ μm^2^/h) compared to untreated cells (2.61·10^4^ μm^2^/h) (Figure 2E).

These results suggest that Chloroquine reduce the collective migration rate, velocity and healing speed compared to untreated cells (Maiques et al., 2018).

### Assessment of Mibefradil Treatment in Individual Cell-Tracking Assay

The *in vitro* individual cell-tracking assay is a method to analyze the migration distance and velocity of single cells independently from cell-cell interactions, either after a drug treatment, in mutant cells, or in siRNA libraries. We studied the effect of T-type calcium channel (TTCC) blocker (Mibefradil) in melanoma individual cell migration.

We assessed the individual cell trajectories in M3 melanoma cells after 20 h treatment with Mibefradil (5 μM). Phase-contrast representative images of Mibefradil treated cells (right panel) and untreated cells (left panel) before (0 h) and after (20 h) live cell imaging analysis are shown (Figure 3A). The representative cell migration trajectories were plotted with a common origin in untreated (green) and Mibefradil treated cells (red) (Figure 3B). Consequently, we quantified the accumulated distance average (μm) (Figure 3C) and velocity (μm/h) (Figure 3D). We observed that untreated cells present a mean of 280.57 ± 42.74 μm and 15.30 ± 2.33 μm/h in accumulated distances and velocity, respectively whereas the treated cells showed a significant reduction (*p* = 0.0074; *p* = 0.0158) in both parameters (148.83 ± 15.42 μm and 8.70 ± 1.61 μm/h, respectively).

These findings revealed that Mibefradil significantly impaired M3 melanoma cells motility in a single cell scale (Maiques et al., 2018).

### Chemoattractant-Driven Cell Migration/Invasion in Transwell Assays

In order to analyze the ability of melanoma single cells to directionally respond to various chemoattractants and treatments (TTCC blockers) we have performed a transwell assay. In this assay, we can evaluate the migration or invasive capacity in cancerous cells, depending on the use of matrigel or not (see **STEPWISE PROCEDURES**).

M3 melanoma cells were seeded in the upper part of the membrane (see scheme in Figure 4A) and treated either with or without FBS (used as chemoattractant) at the upper chamber and/or treated with FBS and Mibefradil at the bottom chambers (see scheme in Figure 4B).

We next investigated melanoma cells migration capacity under the effect of 10% FBS as a chemoattractant and Mibefradil (5 μM) treatment. Total melanoma cells (input, left panels in Figure 4C) and migrated ones (right panels in Figure 4C) were stained with Hoechst, and cell number in each side of the transwell was counted. Quantitative analysis shows that only 17 ± 5.04% of untreated cells migrated without FBS, whereas 10% FBS chemoattraction induced a significant increase in cell migration percentage (48.01 ± 4.98%). To evaluate the effect of TTCC blockers treatment, we compared the untreated M3 cells with 10% FBS (67.22 ± 10.37% cells migrated) with M3 cells treated with Mibefradil and 10% FBS (49.32 ± 6.23% cells migrated). We observed a significant decrease in migration rate when cells are treated with TTCC blockers (*p* = 0.0009). Contrarily, when there is rich media (with 10% FBS) in the upper and lower chamber, there is not significant decrease in migration by Mibefradil treatment (*p* = 0.6358) because there is no chemoattractant gradient to favor migration directionality (Figure 4D).

All these results suggest that FBS chemoattractant increases cell migration capacity, but this effect can be attenuated with Mibefradil treatment, which reduces migration rate by blocking the autophagic flux in M3 melanoma cells (Macià et al., 2015; Maiques et al., 2018).

### Assessment of Spreading Capacity in Melanoma Cells

Another characteristic aspect in melanoma cells behavior studies is the spreading capacity. In melanoma cells, adhesion, cytoskeleton structure, and cell size can be compromised (Salvatierra et al., 2015). We analyzed M3 cell line spreading capacity either treated or not with TTCC blocker Mibefradil.

Spreading assay is schematically described (Figure 5A), where cells were treated for 24 h with Mibefradil (10 μM), were trypsinized and seeded in fibronectin-coated plates. After treatment, fixation and image acquisition, the spreading capacity was quantified using the macro NotSpread&All_.ijm in ImageJ/Fiji (Supplementary Figure 4 and provided in Supplementary Data 3). Roughly, round and refringent cells were annotated as unspread, while darker cells with visible cytoplasm surrounding the nuclei were considered as spread cells. Representative phase-contrast images with spread and unspread untreated M3 melanoma cells (left panel) and Mibefradil-treated cells (right panel) are shown (Figure 5B). The quantitative analysis of spread cells and the effect of the treatment were analyzed, indicating a significant decrease in percentage (*p* > 0.001) of spread cells after Mibefradil treatment (36.25 ± 3.10%) compared to untreated cells (65.68 ± 4.43%) (Figure 5C).

This result indicates that Mibefradil impairs the spreading capacity in M3 melanoma cells.

## Discussion

Determining migratory, adhesion and invasion phenotype of tumor cells and understanding molecular mechanisms is fundamental for novel clinical strategies in cancer diagnosis, prognosis, drug development, and treatment. Metastasis is the main cause of cancer lethality, 90% of deaths from solid tumors can be ascribed to metastatic dissemination (Kramer et al., 2013), and understanding the multi-step migration, adhesion and invasion progress, represents an enormous challenge in cancer treatment (Anderson et al., 2019). Quantification of *in vitro* migration processes in cancer cell lines using time-lapse microscopy can be a crucial tool to study new potential therapeutic anti-cancer drugs, and also to understand basic principles of novel molecular metastatic pathways.

Our *in vitro* step-by-step protocols offer, to a wide range of scientists the capacity to determine and measure an extensive variety of cell motility parameters related to the migration process, such as wound area, velocity, healing speed, front cell velocity, traveled distance, invasion, and spreading rate. Further, these protocols explain an accurate and robust procedure to quantify these parameters using ImageJ/Fiji software.

The main advantages of these *in vitro* assays are that they are relatively easy handling, fast, accurate and with high reproducibility, non-expensive and do not require particularly special equipment. Moreover, they often allow the examination and phenotypic analysis during single cell assay. The migration-related phenotypes determined by these methods may provide useful information about the metastatic potential of the type of cancer studied *in vivo* for the prognosis of the disease.

Still, we have to accept that these methodologies have some limitations, which may be mitigated with specific adjustments. Regarding the wound healing assay, in initial stages of your research you may perform a scratch assay with pipette tip just taking images at initial and end time (in order to screen for several drugs and/or doses). This analysis is simple and gives valuable initial information about cell front migration, but does not provide dynamic information. After this initial screen, we can proceed to study exhaustively the wound closure time and cell front healing speed using the insert and recording the complete time-lapse. Individual cell migration analysis should be used after researchers have evaluated their cell type velocity and have stablished whether they move independently or not, since they exist cell lines that need to form a group to activate migration mechanism (De Pascalis and Etienne-Manneville, 2017). Related to the invasion analysis using the transwell assay, it is essential to check previously if cells are capable to invade the membrane and the Matrigel coating, as there exist cell types which can migrate horizontally very fast but they cannot invade a pore membrane (Trepat et al., 2012). Another phenotype to take into account is whether cells are able to grow vertically; if that is not the case they should not be used in this kind of invasion assays.

Depending on the aim of your research, it may be interesting to make use of some other modifications. For example, the use of fluorescent-labeled cells (either genetically modified to express fluorescent proteins such as GFP fusions or stained with fluorescent cell tracker dyes) will provide an extra layer of relevant biological information by itself (e.g., protein localization, expression levels, metabolic state of certain organelles, etc.), but it will also increase image segmentation efficiency for its further analysis. Nevertheless, we have to be cautious when obtaining fluorescence images, since excitation and/or emitted light could generate photo-toxicity and photo-sensitization in live imaging experiments, thus altering cell behavior and even its morphology (Icha et al., 2017). Besides, the methods described in this protocol may be used as a basis, but in order to deal with fluorescence images some steps should be modified.

The high throughput image data generated by these methods and specially its downstream quantification, represent one of the major limitations in its clinical application. In order to accelerate this step, we wrote two novel macros for running in ImageJ/Fiji program for the quantification of wound healing and spreading assays. These automated methods are not as accurate as the manual ones, but the analysis is done much faster, which enables to quantify a higher amount of images and get more statistically robust data in a fraction of time. Moreover, we specified the ImageJ/Fiji built-in functions that provide an accurate image analyses in single cell and transwell assays.

Altogether, these varieties of assays generate quantitative metrics capable of describing accurately the migratory behavior of cancer cells from different perspectives. We are convinced that the inclusion of these analyses with the corresponding quantitative methods will be a powerful tool for the characterization of cancer metastatic prognosis and for preclinical screening of novel therapeutic drugs designed to impair metastatic progression.

## Author Contributions

JP, RM, AM, and AP conceived and designed this project. JP, CB, DM, OM, PS, AM, and AP performed the experiments and analyzed the results. JP and DM developed the macros. JP, AM, and AP wrote the first draft of the manuscript. All authors contributed to manuscript revision, read and approved the submitted version.

### Conflict of Interest Statement

The authors declare that the research was conducted in the absence of any commercial or financial relationships that could be constructed as a potential conflict of interest.

## Supplementary Material

Supplementary Figure 1 Step-by-step setting of Wound_healing macro for ImageJ/Fiji. **(A)** *Set scale* and *Set measurements* dialog boxes allow adjusting the scale (pixel/μm) and the parameters to measure **(B–D)**. Step-by-step of different settings to determine the measurable area by applying *Find edges, Gaussian blur* (of radius 5 pixels), and *Threshold adjust*. **(E)** Final step and results (area values) of analysis with Wound_healing macro.

Supplementary Figure 2 Screenshots to set step-by-step the Manual Tracking and Chemotaxis tool plugins by ImageJ/Fiji. **(A)** Time-lapse sequence and main window of Manual Tracking with the different parameters to set *time interval, x/y calibration* and *track*. **(B)** Results window after tracking cells at different time-points by Manual Tracking, *track* n°, *slice* n°, *X, Y, distance, velocity* and *pixel value* are shown. **(C,D)** Main window of Chemotaxis tool plugin with *time interval, x/y calibration* and *plot size* and import dataset settings.

Supplementary Figure 3 Setting to count cell number in ImageJ/Fiji. **(A)** Fluorescent image with migrated cells after 20 h in transwell insert (left panel). Nuclei are stained with Hoechst (blue fluorescence). Scale bars, 100 μm. After applying the *Find maxima* parameter (e.g., noise tolerance 5) in ImageJ/Fiji, an automatically cell recount was applied (right panel). **(B)** The same fluorescent image, as in **(A)**, followed by a manual cell recount using the *Cell counter* application in ImageJ/Fiji (right panel).

Supplementary Figure 4 Step-by-step proceeding used in order to count the number of unattached and spread cells in ImageJ/Fiji. **(A)** Brightfield images of the fixed cells are background subtracted and thresholded (using the *Yen method*), and non-attached cell number is counted after a few binary operations, including fill holes and watershed. **(B)** Brightfield images of the fixed cells are background subtracted, edges are found and the edged image is thresholded (using the *Triangle method*), and total cell number is counted after a few binary operations, including fill holes, and watershed.

Supplementary Data 1 Wound_healing.ijm macro code.

Supplementary Data 2 Wound_healing_Choose_Threshold.ijm macro code.

Supplementary Data 3 NotSpread&All.ijm macro code.
