# Supplementary figures and images for: In vitro Cell Migration, Invasion, and Adhesion Assays: From Cell Imaging to Data Analysis
Source: Front Cell Dev Biol. 2019 Jun 14;7:107. doi: 10.3389/fcell.2019.00107 (PMC6587234; doi:10.3389/fcell.2019.00107)

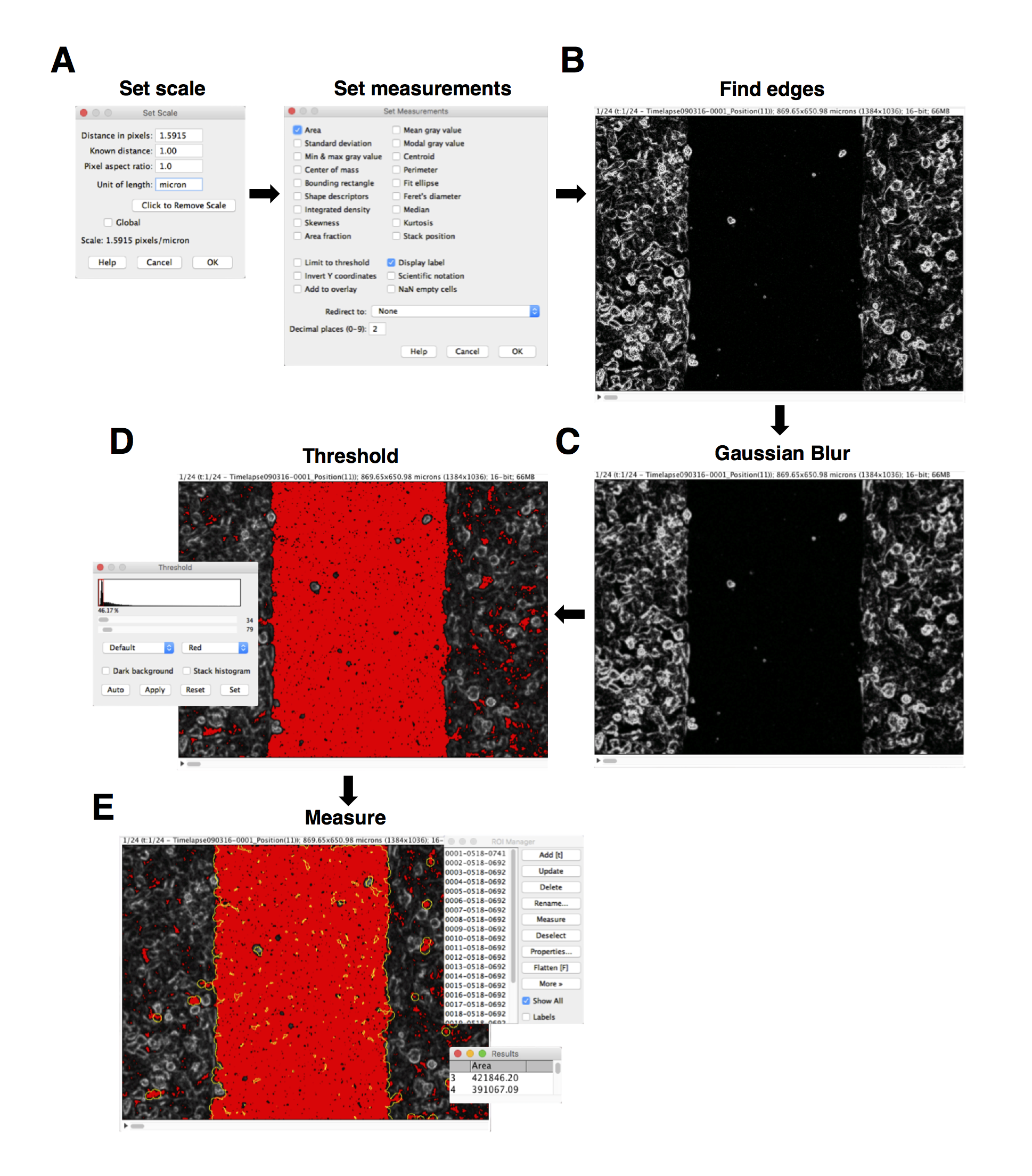

Supplement: Supplementary Figure 1 — Step-by-step setting of Wound_healing macro for ImageJ/Fiji. (A) Set scale and Set measurements dialog boxes allow adjusting the scale (pixel/μm) and the parameters to measure (B–D). Step-by-step of different settings to determine the measurable area by applying Find edges, Gaussian blur (of radius 5 pixels), and Threshold adjust. (E) Final step and results (area values) of analysis with Wound_healing macro. [file Image_1.TIFF]

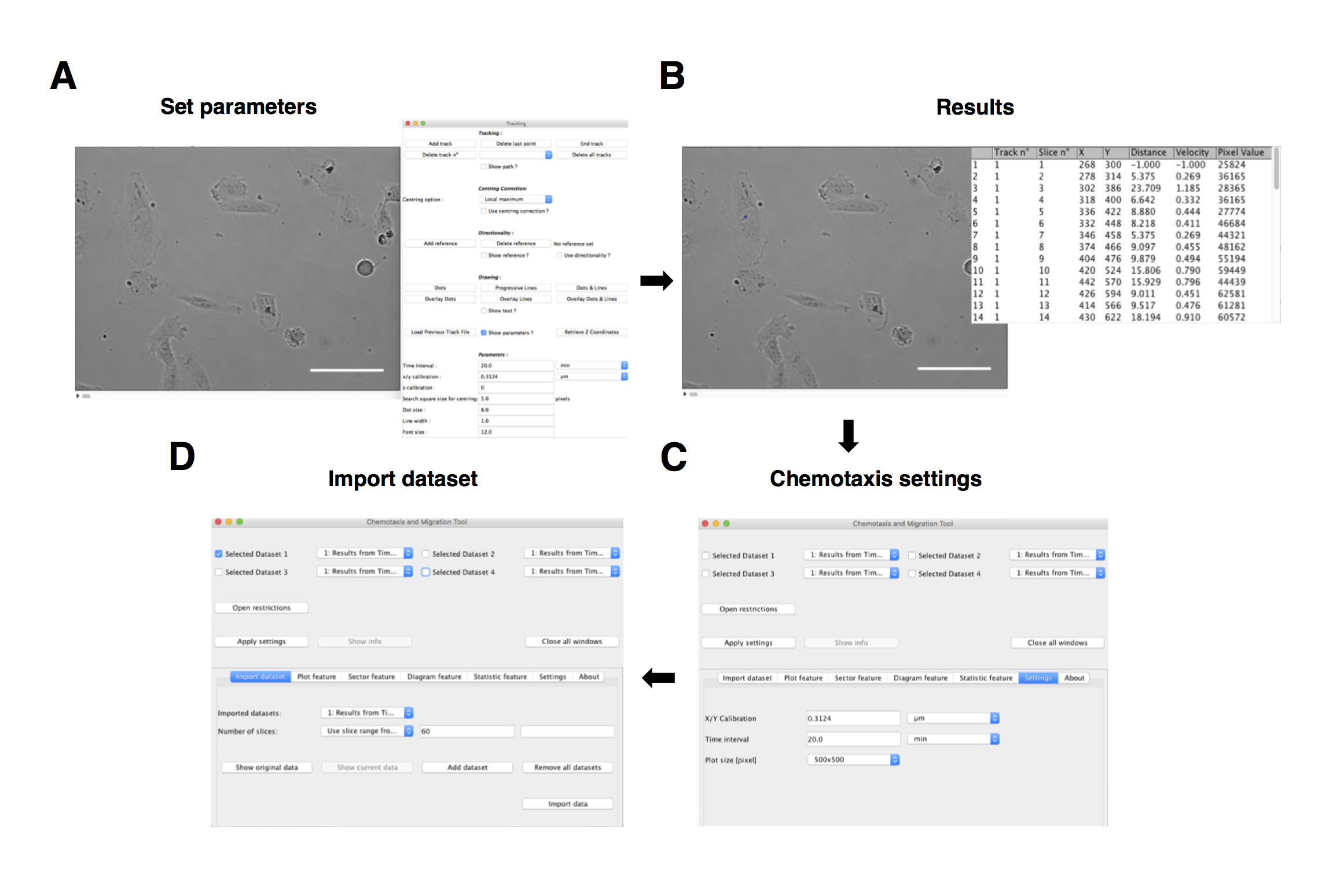

Supplement: Supplementary Figure 2 — Screenshots to set step-by-step the Manual Tracking and Chemotaxis tool plugins by ImageJ/Fiji. (A) Time-lapse sequence and main window of Manual Tracking with the different parameters to set time interval, x/y calibration and track. (B) Results window after tracking cells at different time-points by Manual Tracking, track n°, slice n°, X, Y, distance, velocity and pixel value are shown. (C,D) Main window of Chemotaxis tool plugin with time interval, x/y calibration and plot size and import dataset settings. [file Image_2.TIFF]

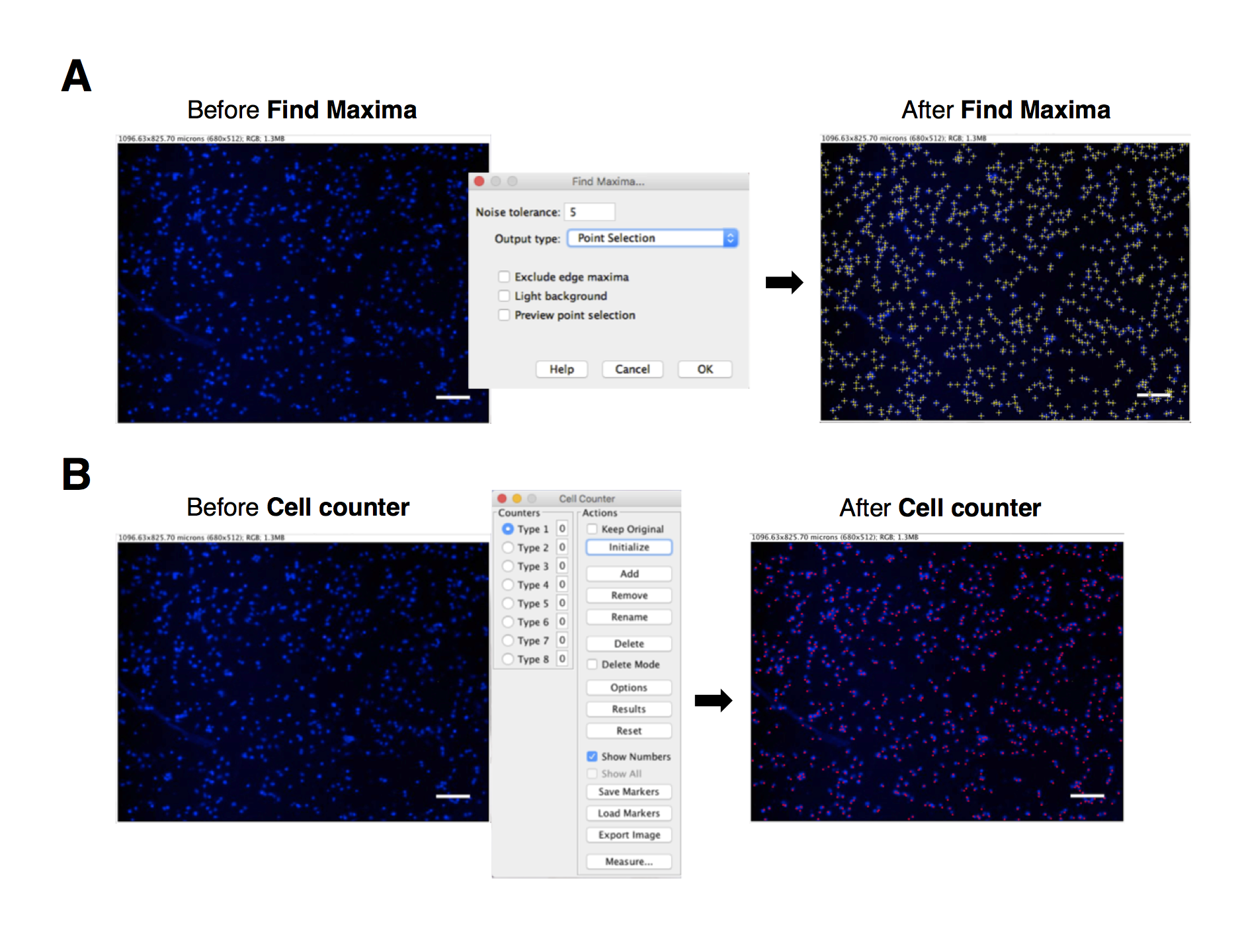

Supplement: Supplementary Figure 3 — Setting to count cell number in ImageJ/Fiji. (A) Fluorescent image with migrated cells after 20 h in transwell insert (left panel). Nuclei are stained with Hoechst (blue fluorescence). Scale bars, 100 μm. After applying the Find maxima parameter (e.g., noise tolerance 5) in ImageJ/Fiji, an automatically cell recount was applied (right panel). (B) The same fluorescent image, as in (A), followed by a manual cell recount using the Cell counter application in ImageJ/Fiji (right panel). [file Image_3.TIFF]
